# Bird sound spectrogram decomposition through Non-Negative Matrix Factorization for the acoustic classification of bird species

**DOI:** 10.1371/journal.pone.0179403

**Published:** 2017-06-19

**Authors:** Jimmy Ludeña-Choez, Raisa Quispe-Soncco, Ascensión Gallardo-Antolín

**Affiliations:** 1 Facultad de Ingeniería y Computación, Centro de Investigación en Electrónica y Telecomunicaciones (CIET), Grupo de Investigación en Ciencia y Tecnología de Materiales (CITEM), Universidad Católica San Pablo, Arequipa, Perú; 2 Department of Signal Theory and Communications, Universidad Carlos III de Madrid, Madrid, Spain; University of South Florida, UNITED STATES

## Abstract

Feature extraction for Acoustic Bird Species Classification (ABSC) tasks has traditionally been based on parametric representations that were specifically developed for speech signals, such as Mel Frequency Cepstral Coefficients (MFCC). However, the discrimination capabilities of these features for ABSC could be enhanced by accounting for the vocal production mechanisms of birds, and, in particular, the spectro-temporal structure of bird sounds. In this paper, a new front-end for ABSC is proposed that incorporates this specific information through the non-negative decomposition of bird sound spectrograms. It consists of the following two different stages: short-time feature extraction and temporal feature integration. In the first stage, which aims at providing a better spectral representation of bird sounds on a frame-by-frame basis, two methods are evaluated. In the first method, cepstral-like features (NMF_CC) are extracted by using a filter bank that is automatically learned by means of the application of Non-Negative Matrix Factorization (NMF) on bird audio spectrograms. In the second method, the features are directly derived from the activation coefficients of the spectrogram decomposition as performed through NMF (H_CC). The second stage summarizes the most relevant information contained in the short-time features by computing several statistical measures over long segments. The experiments show that the use of NMF_CC and H_CC in conjunction with temporal integration significantly improves the performance of a Support Vector Machine (SVM)-based ABSC system with respect to conventional MFCC.

## Introduction

In recent years, the problem of automatically detecting and classifying different species of birds using audio signals (bio-acoustics) has attracted the attention of numerous researchers, especially as a new tool for the remote and non-invasive monitoring of birds populations, allowing researchers to describe the interactions of the species under study, gain new insights into the social dynamics of birds and track factors such as climate change, habitat, and biodiversity [1, 2]. This study addresses the design of an acoustic bird species classification system that is intended to be used to monitor bird populations in Peru as a useful tool for ornithologists who could automatically identify the presence of local bird species, especially in unreachable areas.

In general, the Acoustic Bird Species Classification (ABSC) task can be formulated as a machine learning problem consisting in the following two primary stages: feature extraction (or front-end) and classification (or back-end). The first stage obtains a parametric and compact representation of the audio signals (in this case, birds sounds) that are more appropriate for classification. The purpose of the second one is to determine which bird species corresponds to the analyzed audio signal using a certain decision process. Several front-ends and classifiers have been proposed and compared in the literature for ABSC tasks. Regarding the classification stage, Hidden Markov Models (HMM) [3–5], Gaussian Mixture Models (GMM) [3, 4, 6] and more recently, Support Vector Machines (SVM) [7–9] are worth mentioning.

With respect to the front-end stage, the design of an appropriate parameterization scheme is essential for capturing the specific features of each species because, otherwise, it could cause serious mistakes during acoustic bird species modeling [10]. This paper focuses on the front-end stage, and its primary objective is the design of a suitable feature extraction method for ABSC.

Many state-of-the-art front-ends for audio signals are composed of two primary modules, namely short-time feature extraction and temporal feature integration. In the first one, acoustic coefficients are computed on a frame-by-frame basis (typically, the frame period used for bird audio analysis lasts approximately 5–10 ms) from analysis windows of 20–40 ms. The most frequently used short-time characteristics are the Mel Frequency Cepstral Coefficients (MFCC) [4–8, 10–12]. In the temporal feature integration module, features at larger time scales are extracted by combining the short-time parameters information over a longer time-frame composed of several consecutive frames. The resulting characteristics are often called segmental features and they have been used successfully in several audio-related tasks, such as general audio [13], music genre [14] and acoustic event classification [15–18].

Nevertheless, conventional acoustic features are not necessarily the most appropriate for acoustic bird species classification because most of them have been designed according to the spectral characteristics of speech, which is quite different from the spectral structure of bird sounds [19].

For this reason, it is essential to find a set of features that adequately represent this type of acoustic signals while accounting for the bird sound production system. In recent years, the Non-Negative Matrix Factorization (NMF) algorithm has been used to find a suitable representation of data with satisfactory results in different areas in relation to signal and data processing, such as the following fields: audio and speech [20–22], images [23], electroencephalograms [24, 25], and text mining [26]. NMF is an unsupervised algorithm that engages with a linear representation of data through the decomposition of the non-negative matrix containing these data into a product of two non-negative matrices, with one containing the basis vectors or components of the data and the other one containing the corresponding activation coefficients or gains.

NMF has been previously used to optimize the front-end of audio classification systems. In particular, in our previous work about acoustic event classification [18], NMF was used in the second stage of the front-end (temporal feature integration) for improving the modeling of the dynamic behavior of short-time features. In this case, only the basis vectors provided by NMF were used, and the information contained in the activation coefficients was discarded. By contrast, in this paper, NMF is used to perform a non-negative decomposition of bird sound spectrograms with the aim of optimizing the first stage of the front-end (short-time feature extraction) in a bird sound classification system. In particular, two different alternatives for using NMF in this framework are proposed. In the first, short-time features are deployed from an auditory filter bank, which is trained by means of the NMF algorithm on bird audio spectrograms in an attempt to obtain a better fit between this filter bank and the spectro-temporal characteristics of bird sounds. In the second one, the short-time characteristics are calculated from the activation coefficients matrix resulting from the bird spectrogram decomposition as performed through NMF. Note that in this latter case, the information contained in the NMF activation coefficients is directly incorporated in the short-time features. In both alternatives, the final set of segmental features is obtained by applying a conventional temporal feature integration technique consisting of the computation of several statistics of these improved short-time acoustic parameters over long temporal windows.

## Methods

### Non-Negative Matrix Factorization (NMF)

In this section, we provide a brief description about the mathematical foundations of Non-Negative Matrix Factorization because it provides the background of the two short-time feature extraction schemes proposed in this paper. Given a matrix $V\in R_{+}^{F\times T}$, where each column is a data vector, NMF approximates it as a product of two non-negative low-rank matrices **W** and **H**, such that

$$\begin{array}{c} V\approx \text{WH}, \end{array}$$ (1)

where $W\in R_{+}^{F\times K}$ and $H\in R_{+}^{K\times T}$ and normally *K* ≤ *min* (*F*, *T*). In this way, each column of **V** can be written as a linear combination of the *K* basis vectors (columns of **W**), which are weighted with the coefficients of activation or gains located in the corresponding column of **H**. NMF can be seen as a dimensionality reduction in data vectors from an *F*—dimensional space to a *K*—dimensional space. This finding is possible if the columns of **W** uncover the latent structure in the data [27]. The value of *K* must be selected in advance by accounting for the specific application of NMF.

Factorization is achieved by an iterative minimization of a given cost function such as, for example, the Euclidean distance or the generalized Kullback-Leibler (KL) divergence which is defined as follows:

$$\begin{array}{c} D_{\text{KL}}\left(V∥\text{WH}\right)=\sum_{ij}\left(V_{ij}log\frac{V_{ij}}{{\left(\text{WH}\right)}_{ij}}-{\left(V-\text{WH}\right)}_{ij}\right) \end{array}$$ (2)

The Kullback-Leibler divergence results in a non-negative quantity and is unbounded. In this work, the KL divergence is considered because it has recently been used, with good results, in audio processing tasks such as speech enhancement and denoising for automatic speech recognition [21, 28], feature extraction [22] or acoustic event classification [22, 29]. To find a local optimum value for the KL divergence between **V** and (**WH**), an iterative scheme with multiplicative update rules can be used as proposed in [27] and stated in Eqs (3) and (4),

$$\begin{array}{c} W\leftarrow W⊗\frac{\frac{V}{\text{WH}}H^{T}}{1H^{T}}, \end{array}$$ (3)

$$\begin{array}{c} H\leftarrow H⊗\frac{W^{T}\frac{V}{\text{WH}}}{W^{T}1}, \end{array}$$ (4)

where **1** is a matrix of size **V**, whose elements are all ones, and the multiplications ⊗ and divisions are component-wise operations. NMF produces a sparse representation of the data, reducing redundancy.

As explained in the coming sections, matrix **V** is composed of bird sound spectrograms in our case, so NMF is used to obtain their non-negative decomposition to attempt to discover the primary frequency components of bird sounds.

### Feature extraction for ABSC

The overall feature extraction process for the ABSC task consists of the following three primary stages: syllable segmentation and pre-processing, short-time feature extraction and temporal feature integration.

#### Syllable segmentation and pre-processing

Bird sounds are usually divided into two categories, long-term vocalizations (songs) and short-term vocalizations (calls). Songs are usually related to breeding and territorial defense, while calls have functions as alarms, flight or feeding. In this paper, the separation between these categories is not considered. Bird sounds are structured into hierarchical levels such as phrases, syllables, and elements so that a phrase is formed by a series of syllables, which, in turn, are constructed by elements. In this study, syllables are considered as the fundamental acoustic units, so bird acoustic signals are first segmented into syllables. The method proposed in [30] is adopted for this purpose. This algorithm is based on the assumption that syllables can be adequately modeled as sinusoidal pulses with distinctive amplitude and frequency variations, and they correspond to temporal regions in which the magnitude spectrum is above a predefined threshold. After preliminary experimentation, in our case, this threshold was set to 20 dB because this value minimized the number of audio files in which no syllables were detected. The performance of the segmentation stage itself was not explicitly measured because manual boundaries were not available due to the difficulty of this task, which must be performed by expert ornithologists. Nevertheless, the automatic segmentation stage was fixed in all the experiments that were conducted in the Experiments and results section, so its influence on the classification rate of the whole system is the same for all the evaluated front-ends.

To improve the acoustic classification of bird sounds, it is essential to perform a proper pre-processing step, which consists of filtering the audio signal using a Butterworth pass band filter with a pass band between 1 kHz and 10.5 kHz. This frequency band was chosen by accounting for the spectral region in which the different bird species sounds are concentrated. The filtering process is necessary because many of the audio recordings are contaminated with noise, such as ambient low-frequency noise or sounds with frequencies corresponding to other species.

#### Short-time feature extraction

For the baseline system, the considered short-time acoustic characteristics are the well-known Mel Frequency Cepstral Coefficients. They are extracted on a frame-by-frame basis using a Hamming analysis window that is 20 ms long with a frame shift of 10 ms. After Hamming windowing, an auditory filter bank composed of 40 triangular mel-scaled filters is applied over the spectrogram that is computed by using the Short-Time Fourier Transform (STFT). This filtering process simulates the behavior of the human auditory system, which is known to be more discriminating of low frequencies. Once the log-energies of the outputs of each filter are calculated, a Discrete Cosine Transform (DCT) is applied to decorrelate them. In addition, the log-energy of each frame and the first derivatives (also called Δ features) are computed and added to the cepstral coefficients, yielding to a 13 (or 26 when the first derivatives are used)-dimension short-time feature vector.

Two different alternatives based on NMF for short-time feature extraction are proposed in this work. Both of them have been motivated by the ability of the NMF algorithm to achieve a good representation of sounds, extracting the most relevant and less redundant components and therefore allowing us to distinguish between different bird species sounds. These two feature extraction schemes are detailed in the Parameterization based on NMF auditory filter bank (NMF_CC) and Parameterization based on NMF activation coefficients (H_CC) sections, respectively.

#### Temporal feature integration

To obtain a set of acoustic parameters on a longer time scale, temporal integration is applied to the short-time feature vectors. For this purpose, the sequence of short-time coefficients and their first derivatives (when indicated) contained in each previously extracted syllable is processed by the considered temporal integration technique. In this paper, it consists of the computation of the statistics (the mean, standard deviation and skewness) of the short-time parameters contained in each syllable [17]. These segment-based parameters are the input to the bird sound classifier, which is based on Support Vector Machines.

### Parameterization based on NMF auditory filter bank (NMF_CC)

**Fig 1:**
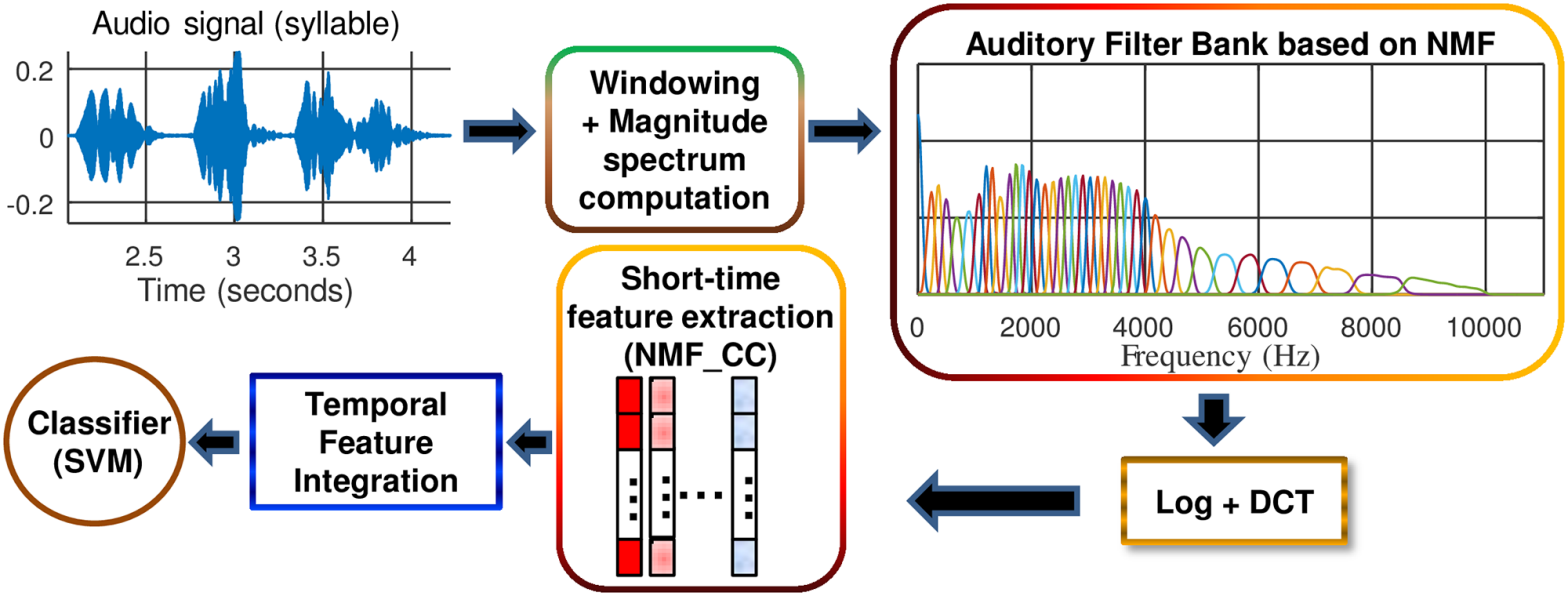
Block diagram of the NMF_CC parameterization. NMF_CC coefficients are computed after applying the Discrete Cosine Transform (DCT) on the audio magnitude spectrum filtered with the NMF-based filter bank. The final set of acoustic features, which is the input of the Support Vector Machine (SVM)-based classifier module, is obtained by performing a temporal feature integration on these short-time parameters.

NMF_CCs are cepstral-like coefficients that are computed using the same procedure as in the case of the conventional MFCC, except that the triangular mel-scaled filters are replaced by an auditory filter bank which is learned in an unsupervised way by applying NMF over the spectrograms of a set of training instances belonging to different bird species. The extraction process is shown in Fig 1.

#### Design of the auditory filter bank using NMF

The primary goal is to develop an unsupervised approach to finding the optimal auditory filter bank in such a way that the resulting cepstral parameters (NMF_CC) adequately represent the different bird species sounds carrying the most significant information about their underlying spectro-temporal structure. This problem can be formulated as the non-negative decomposition of the bird sound spectrograms **V** into their primary components (i.e., into their more relevant frequency bands). In this context, previous works [31] have shown that when Non-Negative Matrix Factorization is applied to spectrograms of speech signals, the resulting decomposition generates a filter bank with remarkable similarities to perceptually motivated auditory filter banks. In this study, we use the same idea for the analysis of bird sounds instead of speech. Along with the rest of the paper, the filter bank obtained by NMF is denoted as **W** to distinguish it from the triangular mel-scaled filter bank **U** used to calculate the conventional MFCC.

In our case, the matrix that will be decomposed is formed by the magnitude spectrograms of the different bird sounds. Because a unique auditory filter bank is learned for all the acoustic classes (bird species) under consideration, matrix **V** consists of the column-wise concatenation of the magnitude spectra that is extracted from the bird sounds contained in the training set of the database. Therefore, the dimension of **V** is (*F* x *n*_*s*_), where *F* is the number of frequency bins and *n*_*s*_ is the total number of frames in the training set.

Once matrix **V** is formed, its corresponding factored matrices (**WH**) are obtained using the learning rules in Eqs (3) and (4). The dimensions of **W** and **H** are, *F* x *K* and *K* x *n*_*s*_, respectively, where *K* is the number of Spectral Basis Vectors (SBV) considered (i.e., the number of filters in the auditory filter bank to be learned). The resulting matrix **W** contains the SBVs, which represent the primary components of the magnitude spectra for the bird acoustic signals because it is verified that **V** ≈ **WH**, and, therefore, they could be interpreted as the filters of the required auditory filter bank.

Note that the NMF-based filter bank **W** is learned during the training stage of the system. In the parameterization process itself, NMF_CCs are computed using this filter bank instead of the conventional mel-scaled filter bank **U**. Finally, the information contained in these short-time features is summarized by means of temporal integration.

### Parameterization based on NMF activation coefficients (H_CC)

In this section, the procedure for feature extraction based on NMF activation coefficients (H_CC) is presented. The primary idea is that during a training stage, it is possible to use NMF for building an acoustic model for each one of the classes under consideration (in this case, the bird species) consisting of the set of spectral basis vectors determined by NMF for this class. After concatenating all these SBVs into a single matrix **W**_*bs*_, the H_CC features of a given bird sound are computed from the activation coefficients **H**_*bs*_ as produced by the application of NMF on its magnitude spectrogram. The hypothesis behind this method is that these **H**_*bs*_-derived coefficients should present good discrimination capabilities, because it is expected that the SBVs of the class to which the bird sound belongs show higher gains than the SBVs of the remaining classes.

#### Learning NMF-based acoustic models

**Fig 2:**
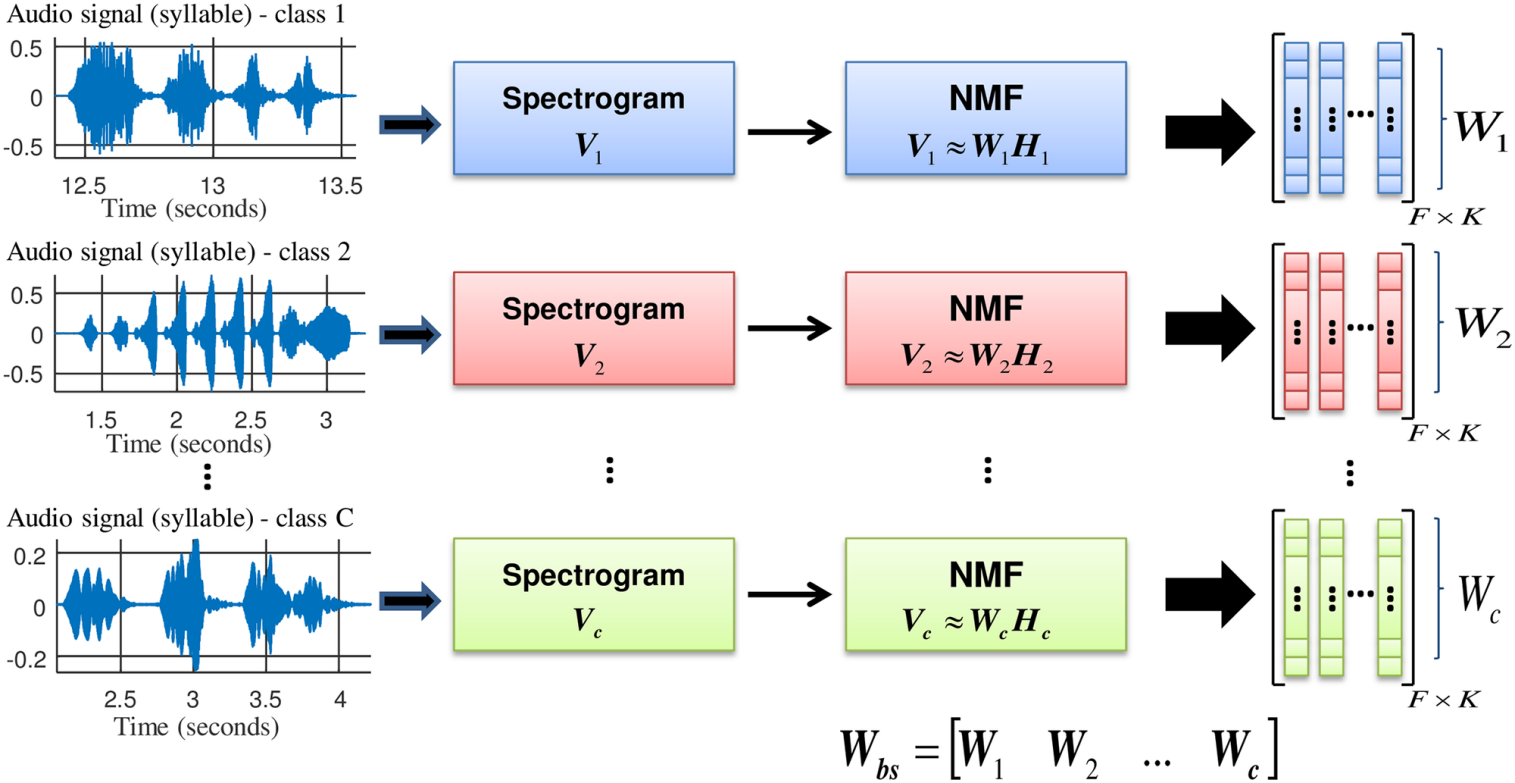
Block diagram of the NMF-based acoustic models building process. The Spectral Basis Vectors (SBVs) of the *i*-th class, **W**_*i*_, are found by applying the NMF algorithm to the training audio data of this class. Finally, the SBVs of all the classes are concatenated to form a single set of SBVs, **W**_*bs*_.

The procedure for obtaining the acoustic patterns is shown in Fig 2, and it is similar to the NMF-based supervised method presented in [28] in which the same idea is utilized to build models of clean speech and noise.

In this work, the acoustic classes are the sounds of the different bird species. Thus, the SBVs of each class are found by applying the NMF algorithm to the training audio data of this class. Specifically, first, the magnitude spectrum of each of the training instances of each bird species is calculated such that the matrices **V**_1_, **V**_2_, …, **V**_*C*_ corresponding to class 1, class 2, …, class *C* are obtained, with *C* being the number of acoustic classes under consideration. Note that each matrix **V**_*i*_ is composed of the concatenation of the magnitude spectrograms of the training instances for the *i*-th class. The KL divergence is then minimized between the magnitude spectra matrix **V**_*i*_ and its factored matrices {**W**_*i*_**H**_*i*_, *i* = 1, …, *C*} using the learning rules given in Eqs (3) and (4).

After this process, the matrix **W**_*i*_ contains the SBVs of the *i*-th class. The dimension of **W**_*i*_ is *F* x *K*, where *F* and *K* are the number of frequency bins used for the computation of spectrograms and the number of spectral basis vectors, respectively. Finally, matrices **W**_*i*_ are concatenated to form a single matrix of SBVs, **W**_*bs*_ with the dimensions *F* x *KC*. These SBVs remain fixed during the feature extraction process itself. Note that contrary to the parameterization method described in the Parameterization based on NMF auditory filter bank (NMF_CC) section, NMF in this case is employed in a supervised fashion as matrices **W**_*i*_ are obtained from previously categorized data.

#### Short-time features derived from NMF activation coefficients

**Fig 3:**
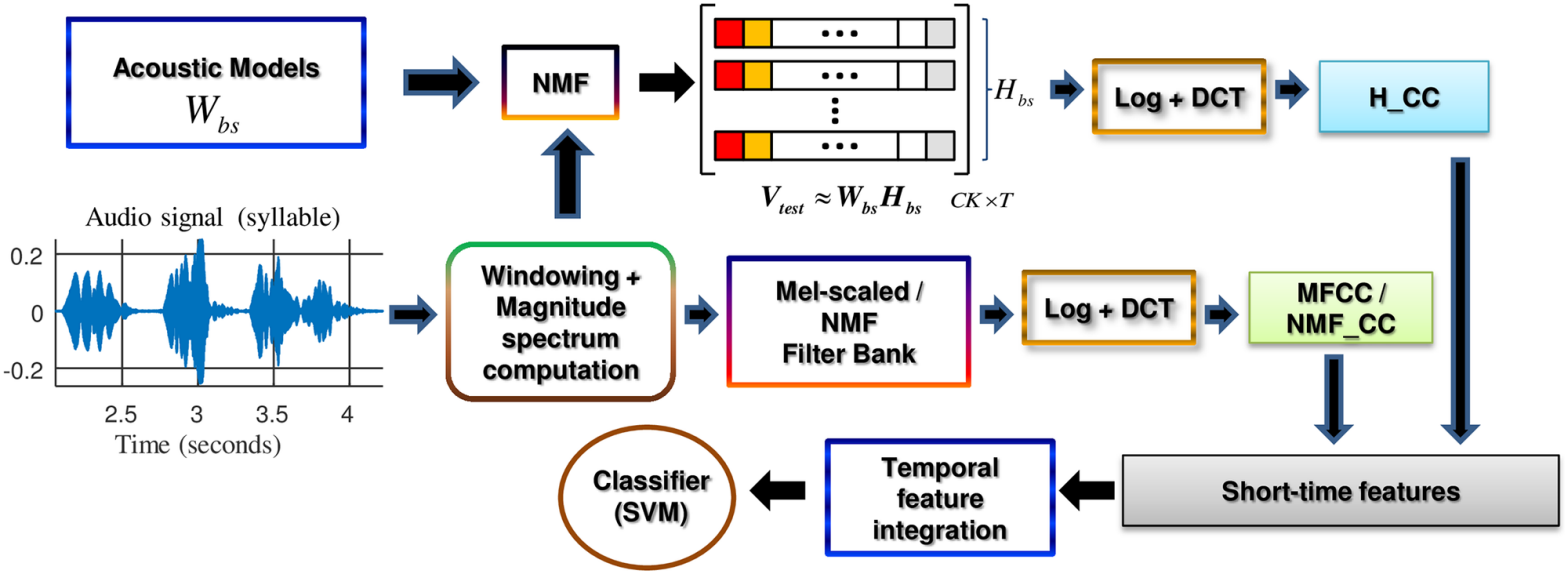
Block diagram of the H_CC (upper part), MFCC/NMF_CC (lower part) feature extraction processes and their combination. The short-time features are obtained by applying the Discrete Cosine Transform (DCT) to the audio magnitude spectrum that is filtered with the conventional mel-scaled (MFCC) or NMF-based filter bank (NMF_CC). The final set of acoustic features, which is the input of the Support Vector Machine (SVM)-based classifier module, is obtained by performing temporal feature integration on the combination of the H_CC and MFCC/NMF_CC short-time parameters.

The upper part of Fig 3 represents the block diagram of the H_CC feature extraction process.

During the parameterization stage, given a test audio signal, its magnitude spectrum **V**_*test*_ is first computed and factored by minimizing the KL divergence between the **V**_*test*_ and (**W**_*bs*_**H**_*bs*_) and then updating only the activation matrix **H**_*bs*_ with the learning rule given in Eq (4). The logarithm of the **H**_*bs*_ values is then computed, yielding to the so-called H_Log coefficients. These parameters are further decorrelated by applying the discrete cosine transform to them, thus generating a set of cepstral-like features, which are denoted as H_CC. Finally, the maximum gain coefficient (G_NMF) is appended to the short-time feature vector. For each frame *t*, this term is calculated as follows:

$$\begin{array}{c} {\left(G_{\_\text{NMF}}\right)}_{t}=\underset{k}{argmax}{\left(H_{bs_{k}}\right)}_{t},k\in \left\{1,...,KC\right\}, \end{array}$$ (5)

where *K* is the number of SBVs per class and *C* is the total number of classes. The use of G_NMF is motivated by the fact that previous studies have shown that it allows for a good characterization of the sound timbre and increases robustness against noise [22, 32].

Once the H_CC short-time parameters are extracted, a temporal integration is applied to obtain segmental-based features as in both conventional MFCC and NMF_CC parameterizations.

### Performance assessment

The performance of the proposed front-ends is measured in terms of the accuracy or classification rate (i.e., the percentage of audio files that are correctly classified). Classification rates obtained by the different parameterization schemes and the corresponding significant differences are computed via a linear mixed model [33]. This model allows us to study the dependence of the accuracy on the parameterization in use, considering the random effects caused by the composition of the database with regards to the 12 bird species included, as described in the Database section, and the adopted experimental protocol, which consists of a *K*-fold cross-validation with *K* = 6, as described in the Experimental setup and baseline system section. The implementation of the linear mixed model was performed by using the *lme4* package [34] for R [35] and the formula shown in Eq (6),

$$\begin{array}{c} \text{Accuracy}∼\text{Parameterization}+(1|\text{Species})+(1|\text{Fold}) \end{array}$$ (6)

which indicates that the parameterization is considered as a fixed effect and the bird species and fold are taken into account as random effects. The model was fitted by a Restricted Maximum Likelihood Estimation (REML) using 792 observations (11 parameterizations x 12 bird species x 6 folds). Statistical significances values were obtained by using the Satterthwaite approximation as implemented in the R *lmerTest* package [36]. The degrees of freedom was 765. The *R*^2^ value was computed by following the method proposed by [37], using the R *sjstats* package [38].

The summary of the model is presented in the Results section. For the fixed effects, coefficients (i.e., accuracy estimates), standard errors and t-values, as well as statistical significance, are shown. For the random effects, the variances and standard deviations are reported. The intercept corresponds to the baseline parameterization MFCC.

## Experiments and results

### Database

The system developed in this work is intended for monitoring bird populations in Peru. To the best of our knowledge, there is no a specific database for this purpose. For this reason, an ad hoc database was created from audio files obtained from the Xeno-canto website [39], which contains real-field recordings of different bird species sounds. Among all the species contained on this website, some of them were selected according to the following two criteria: they had to be resident or migratory bird species in Peru, and the number of recordings had to be large enough to allow for reliable experimentation. Twelve bird species fulfilled these two requirements, so they were ultimately chosen. The final database used for the experiments is available in [40] and, in summary, it consists of a total of 1,316 instances of sounds belonging to the following 10 bird species resident in Peru: *Aramides cajanea*, *Coereba flaveola*, *Colibri thalassinus*, *Crypturellus cinereus*, *Crypturellus obsoletus*, *Crypturellus soui*, *Crypturellus undulatus*, *Lathrotriccus euleri*, *Rupornis magnirostris* and *Synallaxis azarae* and the 2 migratory bird species: *Piranga olivacea* and *Piranga rubra*, for a total of 12 different bird species.

All these species are distributed in the jungle region of Peru; some of them can also be found on the north coast of Peru (*Coereba flaveola*), while *Synallaxis azarae* and *Colibri thalassinus* have a more restricted distribution covering the Yunga region (mountain forest). Readers who are interested in distribution maps of these bird species are referred to [41].

The composition of the whole database is shown in Table 1. All the sounds were converted to mp3 format and sampling frequency (22.05 kHz).

**Table 1:** Composition of the database used in the experiments. The number of audio files and syllables per bird species are indicated.

Class	Bird species	No. of audio files	No. of syllables
1	*Aramides cajanea* [ac]	64	3098
2	*Coereba flaveola* [cf]	201	5039
3	*Colibri thalassinus* [ct]	131	4541
4	*Crypturellus cinereus* [cc]	94	1200
5	*Crypturellus obsoletus* [co]	87	1518
6	*Crypturellus soui* [cs]	100	1012
7	*Crypturellus undulatus* [cu]	68	659
8	*Lathrotriccus euleri* [le]	120	1723
9	*Piranga olivacea* [po]	64	1273
10	*Piranga rubra* [pr]	72	1790
11	*Rupornis magnirostris* [rm]	181	2802
12	*Synallaxis azarae* [sa]	134	3181
Total		1,316	27,836

### Experimental setup and baseline system

Since this database is too small to achieve reliable classification results, a *K*-fold cross-validation was used to extend it artificially and the results were averaged afterward. *K* was set to 6 to achieve a trade-off between the amount of training data necessary to model the different acoustic classes adequately and the amount of testing data necessary for obtaining results with low variance between folds. Specifically, the database was split into six disjointed balanced groups so that one different group was kept for testing in each fold, while the remaining ones were used for training.

The acoustic bird species classification system is based on a one-against-one SVM with a Radial Basis Function (RBF) kernel on normalized features [16, 17]. The SVM-based classifier module was developed using the LIBSVM software [42]. Concerning SVM training, for each one of the sub-experiments, a 5-fold cross validation was used to compute the optimal values of the RBF kernel parameters. In the testing stage, as the SVM classifier was fed with segmental features computed over windows corresponding to the syllables extracted from each audio recording in the segmentation stage, the classification decisions were made at the syllable level. To obtain a decision for the whole instance, the classifier outputs of the corresponding syllables were integrated using a majority voting scheme in such a way that the most frequent label was finally assigned to the whole recording [43].

The different parameterization schemes proposed in this work were implemented in the programming language MATLAB [44]. For the baseline one (MFCC), the speech processing toolbox for MATLAB Voicebox [45] was used. Extensive preliminary experimentation was performed to select their configurations. In particular, several settings relating to window sizes, frame periods and the number of cepstral coefficients were tested, although no important differences in performance were found between them. Finally, the primary details of the final configuration used in the experiments are described below:

- To extract all the short-time features, audio signals are analyzed every 10 ms using a Hamming window of 20 ms.
- The baseline parameterization consists of 12 conventional MFCC (*C*_1_ to *C*_12_) plus the log-energy of each frame. The suffix “+ Δ” indicates that the corresponding first derivatives are also computed and added to these coefficients.
- In the case of the NMF_CC parameterization, 12 cepstral-like features (*C*_1_ to *C*_12_) are computed and the log-energy of each frame is appended to the acoustic vector. In addition, the corresponding first derivatives are appended when indicated with the suffix “+ Δ”.
- In MFCC, the auditory filter bank consists of 40 filters. In the NMF_CC approach, the number of filters in the filter bank to be learned (i.e., the number of SBVs considered) is also *K* = 40.
- In the H_CC parameterization, the number of H_Log features is 48 because the number of spectral basis vectors per class is *K* = 4 and the number of classes corresponding to the different bird species is *C* = 12.
- For the H_CC features, 13 cepstral-like coefficients (*C*_1_ to *C*_13_) are extracted from the 48 H_Log above. The term “+ G_NMF” indicates that the NMF maximum gain term is calculated and added to these coefficients.
- In all cases (MFCC, NMF_CC and H_CC), a temporal integration is applied, yielding to a set of segmental features which consist of statistical measures (the mean, standard deviation and skewness) of the short-time coefficients under consideration.

With respect to the application of NMF to the design of the filter bank **W** in the NMF_CC parameterization (see Design of the auditory filter bank using NMF section) and for the building of the acoustic models in the H_CC parameterization (see Learning NMF-based acoustic models section), in all folds, NMF was initialized by generating 10 random matrices (**W** and **H**) so that the factorization with the smallest Euclidean distance between **V** and (**W** **H**) was chosen for initialization. These initial matrices were then refined by minimizing the KL divergence using the multiplicative update rules given in Eqs (3) and (4) with a maximum of 200 iterations.

### Results

The summary of the linear mixed model that measures the effect of the parameterization used on the classification rate is presented in Table 2. As can be observed the accuracy achieved by MFCC features (intercept) is 69.63%. The remaining coefficients of the fixed effects can be interpreted as the quantity that must be added to the coefficient of the intercept to obtain an accurate estimate for each parameterization. For example, when using LDA1 features, the classification rate decreases by 12.64%, i.e., its accuracy is 56.98% (69.62%—12.64%).

**Table 2:** Fixed and random effects for the linear mixed model that measures the effect of the parameterization used on the accuracy. The front-ends under consideration are MFCC (intercept), LDA1, LDA2, NMF_CC, H_CC + G_NMF and the combinations MFCC + H_CC + G_NMF and NMF_CC + H_CC + G_NMF. The inclusion of the first derivatives is indicated with the suffix “+ Δ‘”. For the fixed effect (parameterization), the classification rate ([%]) estimates, standard errors and t-values are shown. The statistical significance at *p* < 10^−4^ is marked with ***, *p* < 10^−3^ is marked with ** and *p* < 0.05 is marked with *. For the random effects (bird species and fold), variances and standard deviations are reported.

**Fixed effects**:			
	**Estimate**	**Std**. **Error**	**t value**
Intercept	69.623	4.633	12.800 ***
LDA1	−12.636	1.681	−7.519 ***
LDA2	−3.433	1.681	−2.043 *
NMF_CC	1.897	1.681	1.129
H_CC + G_NMF	−3.141	1.681	−1.869
MFCC + Δ	3.070	1.681	1.827
NMF_CC + Δ	5.963	1.681	3.549 ***
MFCC + H_CC + G_NMF	4.070	1.681	2.422 *
NMF_CC + H_CC + G_NMF	4.280	1.681	2.547 *
MFCC + H_CC + G_NMF + Δ	4.393	1.681	2.614 **
NMF_CC + H_CC + G_NMF + Δ	6.119	1.681	3.641 ***
**Random effects**:			
**Groups**		**Variance**	**Std**. **Dev**.
Species		236.884	15.391
Fold		1.871	1.368
Residual		101.668	10.083
Number of obs: 792, groups: Species, 12; Fold, 6			

As a random effect, “Fold” was not significant, because it has a standard deviation near zero, so it is possible to deduce that this random effect has no impact on the model. However, the “Species” random effect has a standard deviation of 15.39, which shows that there is important variability in the system accuracy due to the type of bird species that is recognized. In other words, this result suggests that there are some bird species that are more difficult to classify correctly than others. In the Discussion section, we analyze this issue in more depth. Finally, “Residual” accounts for the variability that is not due to either folds or species. In this case, it could be related to several uncontrolled characteristics in the recordings such as, for example, the presence of environmental noise or sounds produced by other animals. We observe an *R*^2^ value of 0.7167 for the model. From the whole variance explained by the model, 19.71%, 0.15% and 8.46% correspond to the random effects “Species”, “Fold” and “Residual”, respectively.

Apart from the results achieved by MFCC (baseline) and the proposed front-ends, for comparison purposes, Table 2 also contains the accuracies attained by MFCC with first derivatives (MFCC + Δ) and the two methods proposed in [11] (rows labeled “LDA1” and “LDA2”). In LDA1, the short-time acoustic vectors are MFCC plus the log-energy (as in our baseline parameterization); however, instead of considering the corresponding statistics, each syllable is represented by a set of characteristic vectors that is obtained in the training stage by using a clustering algorithm (in particular, the so-called progressive constructive clustering). In addition, Linear Discriminant Analysis (LDA) is further applied to reduce the vector dimensionality. LDA2 is similar to LDA1 with a difference in that the standard deviation and skewness are added to the feature vectors prior to the application of LDA. In both cases, the optimal number of parameters was found to be 18. As shown, the classification rate achieved by LDA1 is considerably lower than the ones obtained using the other methods. LDA2 clearly outperforms LDA1, suggesting that better parametric representations are obtained when the standard deviation and skewness are included in the feature vectors. In any case, conventional MFCC improves the performance of the system significantly in comparison to both LDA1 and LDA2. Regarding the parametrization MFCC + Δ, it increases the accuracy of the system by approximately 3% absolutely with respect to MFCC, although this difference is not statistically significant.

Regarding the comparison between MFCC and NMF_CC-based front-ends, NMF_CC and NMF_CC + Δ achieve a relative error reduction with respect to MFCC of approximately 6.25% and 19.63%, respectively, and this latter result is statistically significant. These results suggest that the filters automatically learned by the NMF algorithm are better suited to model the bird vocal production than the mel-scaled filter bank, which is a better fit for the human production and auditory system.

The parameterization H_CC + G_NMF performs worse than MFCC, although the difference is not statistically significant. Nevertheless, our hypothesis is that because the extraction procedure for MFCC/NMF_CC and H_CC characteristics are quite different, they may convey complementary information in such a way that their combination could provide improvements in the classification rate in comparison to the use of the individual sets of features. To gain insight into this possibility, we performed several experiments with different combinations of these parameters, following the scheme shown in Fig 3.

According to the results shown in Table 2, the combinations MFCC + H_CC + G_NMF and MFCC + H_CC + G_NMF + Δ outperform the baseline system (MFCC), showing relative error reductions of 13.40% and 14.46%, respectively. Similarly, there is an improvement in the performance of the combined systems when the filter bank learned by NMF is used in the calculation of cepstral coefficients instead of the mel-scaled filter bank. In fact, both NMF_CC + H_CC + G_NMF and NMF_CC + H_CC + G_NMF + Δ produce noticeable decreases in the number of misclassifications in comparison to MFCC. In all these cases, the differences in performance with respect to MFCC are statistically significant.

**Fig 4:**
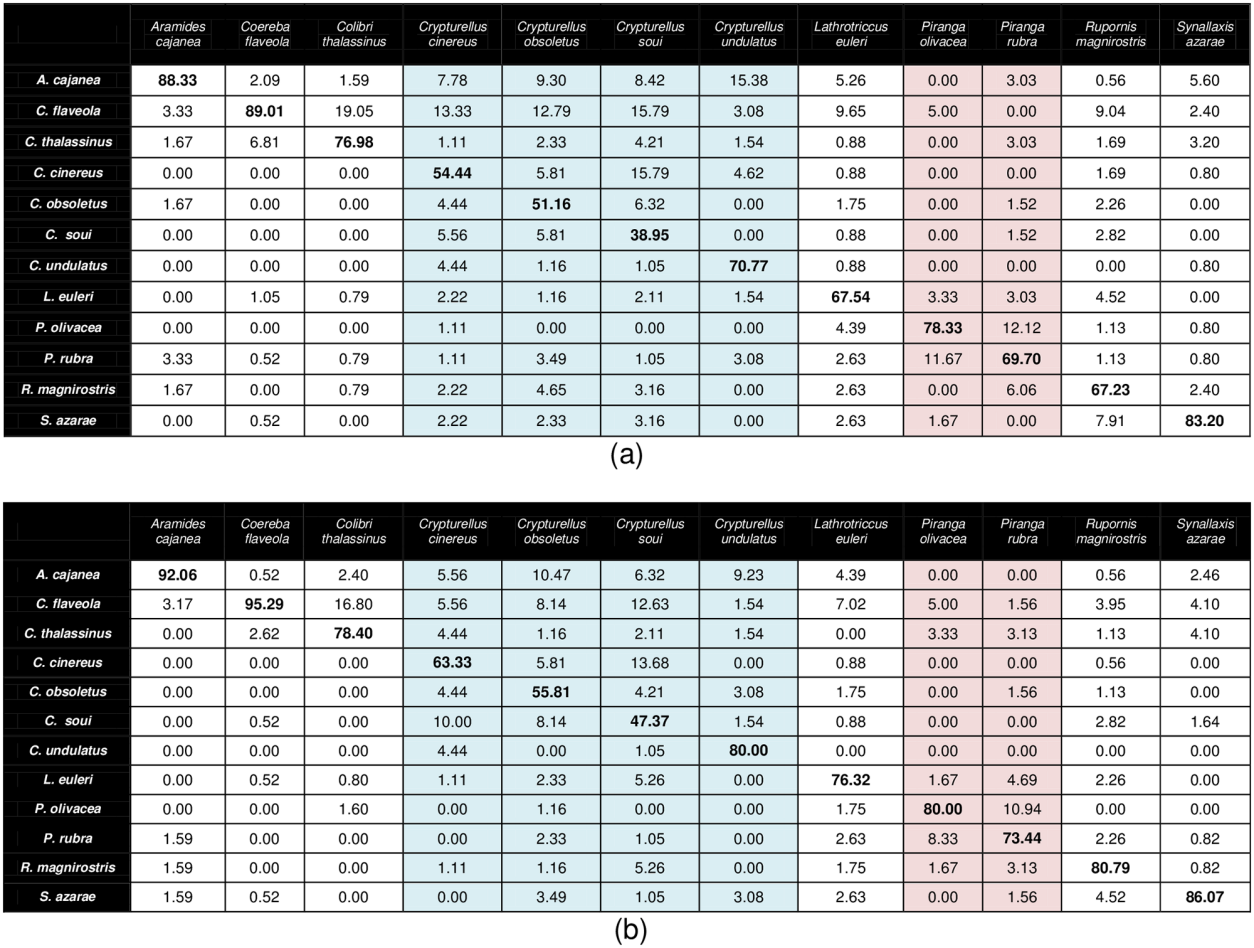
Confusion matrices [%] for two parameterization schemes: (a) MFCC (baseline); (b) NMF_CC + H_CC + G_NMF + Δ (proposed combination). The columns and rows correspond to the correct class and the hypothesized one, respectively. Different colors indicate the audio recordings belonging to species of the same genus.

In summary, the best accuracy attained here is 76.04% which is obtained with the combination NMF_CC + H_CC + G_NMF + Δ and represents a relative error reduction of 20.14% with respect to MFCC. Fig 4(a) and 4(b) show the confusion matrices produced by MFCC and NMF_CC + H_CC + G_NMF + Δ, respectively. In both tables, the columns correspond to the correct class, while the rows are the hypothesized one, and the values within them are calculated by averaging the 6 sub-experiments. Different colors indicate that audio recordings belong to species of the same genus. In particular, light blue and light red indicate Peru native and migratory bird species of the same genus, respectively. As can be observed, for all the acoustic classes, the classification rates achieved by the proposed combination are higher than those obtained by MFCC.

### Execution time

This section is devoted to the comparison of the processing time required by the primary parameterizations proposed in this paper. Although the corresponding implementations have not been explicitly optimized for speed, the intention of this analysis is to determine if the different front-ends could be used in practical applications with a reasonable execution time.

Table 3 shows the total execution time required for the feature extraction process of 1,106 audio files (23,513 frames) for MFCC, NMF_CC and H_CC + G_NMF individual parameterizations and the combination of MFCC with H_CC + G_NMF and NMF_CC with H_CC + G_NMF. Note that the quantities shown in Table 3 do not include the time required for the computation of the first derivative features because it is negligible. The average execution times per frame (recall that each frame corresponds to 10*ms*) and the number of features for each parameterization are also indicated. The comparison was performed on a PC equipped with an Intel(R) Core (TM) i7-4790 processor at 3.60 GHz and a Windows 7 Ultimate 64-bit operating system.

**Table 3:** Execution time (s), average execution time per frame (ms) and number of features for the MFCC, NMF_CC and H_CC + G_NMF parameterizations and their combinations.

Parameterization	Total execution time (s)	Average execution time per frame (ms)	Number of features
MFCC	463.72	19.72	13
NMF_CC	470.26	20.00	13
H_CC + G_NMF	590.02	25.09	14
MFCC + H_CC + G_NMF	1053.74	39.72	27
NMF_CC + H_CC + G_NMF	1060.28	45.09	27

As shown, MFCC has the lowest execution time in comparison to the other front-ends. Nevertheless, the time required by NMF_CC is very similar. This is because, in this case, NMF is only performed once during a previous, independent step in which the optimized filter bank for all the acoustic classes is learned. The computational cost of H_CC + G_NMF is approximately 1.25 times greater than that of MFCC and NMF_CC. This increase is due to the iterative nature of the NMF algorithm, which, in this case, is applied within the parameterization process itself.

As expected, the processing times required by the combinations MFCC + H_CC + G_NMF and NMF_CC + H_CC + G_NMF are 2 and 2.25 times greater, respectively, than that of the baseline parameterization. In addition, the feature dimensions of the combinations are approximately double those of the individual parameterizations. Because there is a trade-off between the classification rate and computational load, in practical applications in which the latter one is critical, NMF_CC + Δ features should be preferred. Otherwise, the combined parameterization NMF_CC + H_CC + G_NMF + Δ should be employed. In any case, all these parameterizations could be used for the off-line processing of audio recordings with an allowable delay.

## Discussion

In this section, we analyze the primary factors that explain the performance of the MFCC and NMF-based front-ends for the ABSC task, which are the capability of each parameterization for achieving an adequate representation of bird sounds, the acoustic similarity between sounds belonging to different species and the presence of noise.

The primary idea behind this work is that NMF provides a better parametric representation of bird sounds than the MFCC approach. To illustrate the validity of this hypothesis, we compare the spectral components used in the MFCC computation with the spectral basis obtained by the two NMF-based front-ends proposed in this paper.

**Fig 5:**
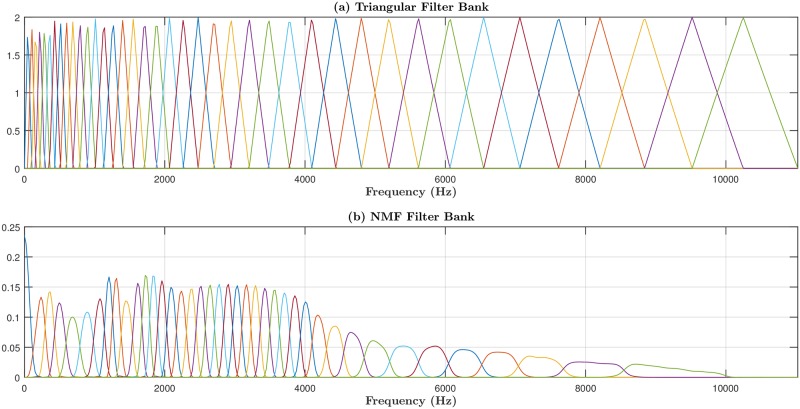
Frequency responses of the auditory filter bank used in the feature extraction process: (a) Triangular mel-scaled filter bank (U); (b) Filter bank determined by NMF (W). To improve the readability of the figures, different colors have been used to represent adjacent filters.

In the first approach, NMF_CC, a common filter bank for all bird species, is learned by using NMF. Fig 5(b) represents this filter bank (**W**) obtained on a single fold for *K* = 40 filters, whereas the conventional mel-scaled filter bank (**U**) is depicted in Fig 5(a). It can be observed that in **W**, a great amount of narrow filters is concentrated in the range between 1 kHz and 5 kHz, suggesting that this frequency band is more relevant to bird sound production. For frequencies greater than 5 kHz, the filter distribution is similar to that of the triangular filter bank, whereas the band below 1 kHz has fewer filters with larger bandwidths. It is worth mentioning that this filter distribution does not differ very much between folds. The resulting NMF filter bank indicates that the spectral nature of bird sounds is quite different from speech spectra, in which most of the relevant components are concentrated in low frequencies. Therefore, it is possible to deduce that NMF_CC is more suitable for the ABSC task than the conventional MFCC.

**Fig 6:**
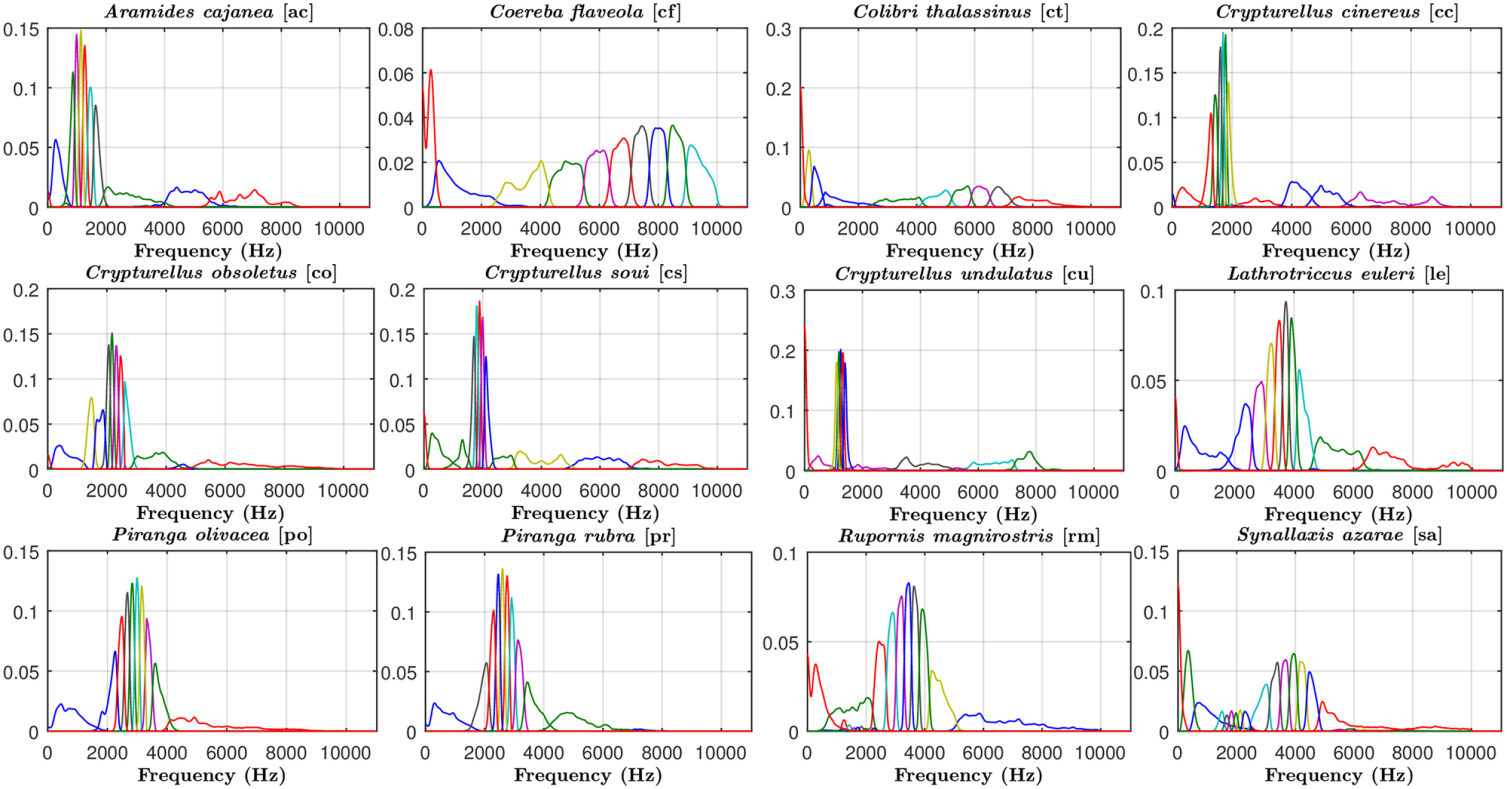
Spectral Basis Vectors (SBVs) for the twelve bird species sounds. To improve the readability of the figures, different colors have been used to represent the adjacent spectral basis vectors.

In the second approach, H_CC, NMF is used to determine the SBVs of each specific bird species, which are represented in Fig 6. Note that although the results of the H_CC front-end presented in the Results section correspond to *K* = 4, we have used *K* = 10 in this discussion to allow for a better graphical representation of the SBVs. As shown here, the spectral contents of bird sounds vary widely between species, presenting generally relevant components in medium-high frequencies. Because these SBVs provide more detailed information about the spectral nature of the different species, it can be argued that H_CC constitutes a better parametric representation for bird sounds than MFCC. However, results show that the performance of H_CC is worse than that of MFCC (although the differences are not statistically significant). This behavior likely arose because of the acoustic similarity between classes, resulting in similar SBVs among bird species (Fig 6), especially those that are closely related (e.g. *Piranga olivacea* and *Piranga rubra*). Confusions between these species were more likely to occur.

**Fig 7:**
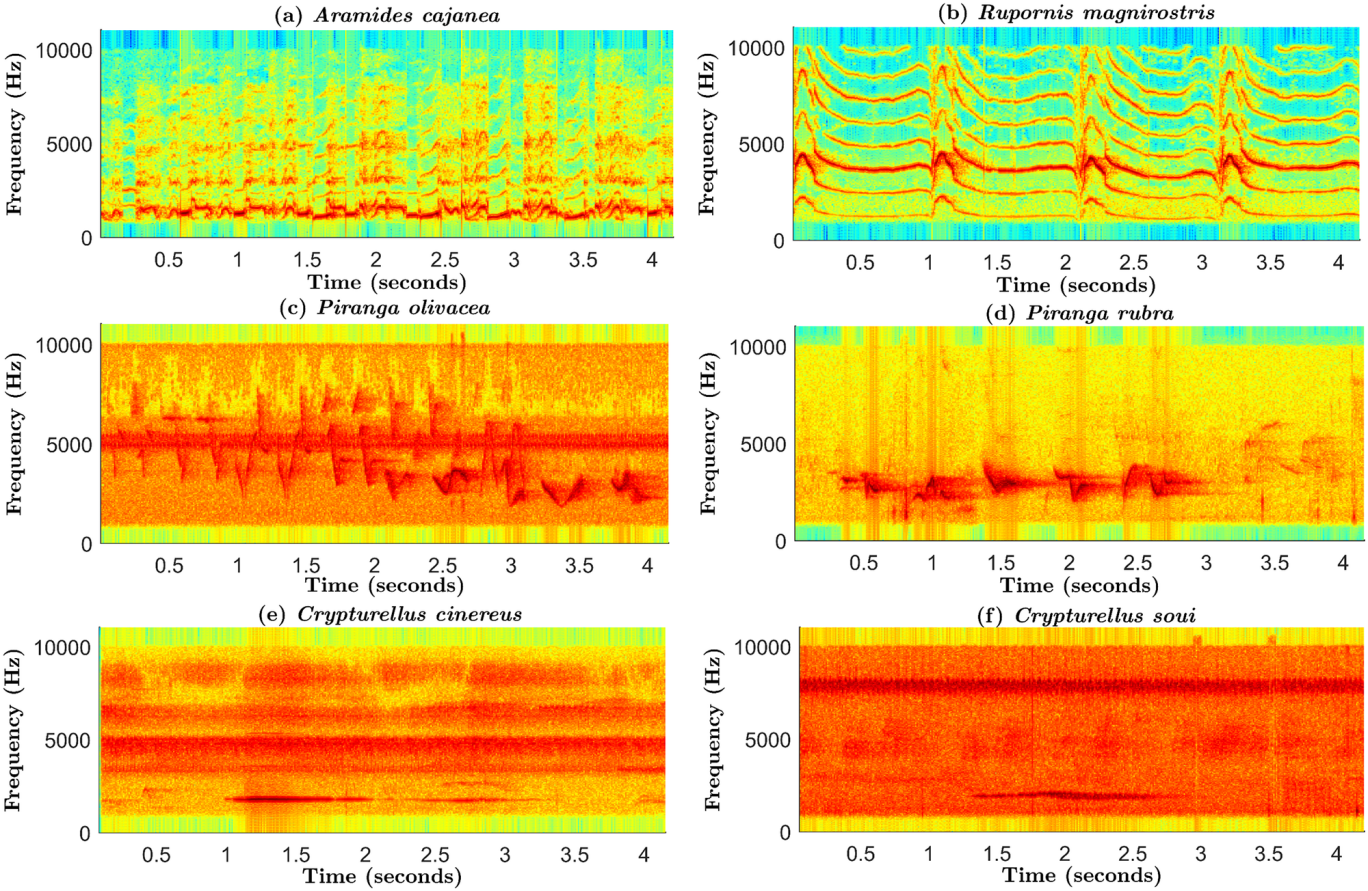
Examples of vocalization spectrograms from the following different bird species: (a) *Aramides cajanea*; (b) *Rupornis magnirostris*; (c) *Piranga olivacea*; (d) *Piranga rubra*; (e) *Crypturellus cinereus*; and (f) *Crypturellus soui*. The two first examples illustrate that, although sounds of some species present an important content in low frequencies as in the case of speech (a), in general, their spectral characteristics are very distinct from the speech spectra (b); (c) and (d) show the acoustic similarity between sounds of different species, which may produce errors in the classification process; and (e) and (f) contain examples of very noisy spectrograms, which are very likely to be misclassified.

Nevertheless, the combination NMF_CC + H_CC + G_NMF + Δ achieves the best accuracy. In the following, we carry out a detailed comparison between the performance of this front-end and MFCC, by analyzing their respective confusion matrices (Fig 4) and several examples of vocalization spectrograms from different bird species (Fig 7).

Although NMF_CC + H_CC + G_NMF + Δ outperforms MFCC for all species (Fig 4), MFCC achieves good results with certain classes (e.g. *Aramides cajanea*). This is possibly due to the “speech-like” characteristics of these sounds, as can be observed in Fig 7(a). This example was correctly recognized by both front-ends. For species whose spectral structure is more different from speech, the proposed combination is better than MFCC, with the differences being especially noticeable for *Crypturellus cinereus*, *Crypturellus soui*, *Crypturellus undulatus*, *Lathrotriccus euleri* and *Rupornis magnirostris*. Fig 7(b) contains a sound spectrogram belonging to this latter species, which was misclassified by MFCC but correctly recognized by the proposed front-end.

In both parameterizations, the acoustic similarity between classes is an important source of errors. Fig 7(c) contains a *Piranga olivacea* spectrogram which was misclassified as *Piranga rubra* by both front-ends, and Fig 7(d) is an example of the opposite case. The level of confusability for NMF_CC + H_CC + G_NMF + Δ is lower than for MFCC (e.g. the percentage of confusions between *Coereba flaveola* and *Colibri thalassinus* decreased from 6.81% with MFCC to 2.62% with the proposed front-end).

The presence of noise or other nature sounds (e.g. crickets) is also a cause of errors. In these conditions, the performance of the syllable detection stage might be negatively affected, which in turn might degrade the accuracy of the classifier (which is also directly affected by noise). Fig 7(e) and 7(f) contain examples of highly noisy spectrograms, which were incorrectly classified by both parameterizations. We have observed that, in general, audio recordings belonging to *Crypturellus cinereus*, *Crypturellus obsoletus* and *Crypturellus soui* are very noisy. This circumstance, together with their pronounced acoustic similarity, produced that these three species were the more difficult ones to classify. In any case, NMF_CC + H_CC + G_NMF + Δ outperforms MFCC for these species, suggesting its robustness to noisy conditions.

## Conclusions

In this paper, a new front-end for acoustic bird species classification whose design incorporates information about the specific spectro-temporal patterns of bird sounds is proposed. It presents a modular structure consisting of the following three different stages: pre-processing, short-time feature extraction and temporal feature integration.

The primary focus of this paper is on the short-time feature extraction module, in which two new parameterization schemes based on the non-negative decomposition of bird sound spectrograms through the application of the NMF algorithm are proposed. In the first scheme (NMF_CC), cepstral-like coefficients are calculated by replacing the conventional triangular mel-scaled auditory filter bank with a NMF-based filter bank. In particular, NMF is used for the unsupervised learning of this auditory filter bank, so that the resulting filters are perceptually motivated according to the bird vocal production system. In the second scheme (H_CC), short-time acoustic characteristics are derived from the NMF activation coefficients. In both cases, the frame-by-frame features are finally combined at a larger temporal scale through a temporal integration process in which statistical measures of these parameters (the mean, standard deviation and skewness) are computed over segments of syllable duration.

The whole front-end has been tested on an SVM-based ABSC system, and the results have been analyzed by using a linear mixed model in which the parameterization is considered as a fixed effect and the bird species and fold are taken into account as random effects. With the first parameterization, the best performance is achieved when NMF_CC and their first derivatives are considered. The results show that the filters learned by NMF are best suited for modeling the bird vocal production mechanism in comparison to the mel-scaled filter bank. In the second parameterization, H_CC by itself does not outperform MFCC; however, its combination with MFCC produces better results than either of the individual feature sets. These classification rates are further improved when the first derivatives are also included and the MFCC are substituted with NMF_CC in the combination. In this case, a relative error reduction with respect to the conventional MFCC system of approximately 20.14% is achieved, and this difference in performance is statistically significant.
